# A novel decrystallizing protein CxEXL22 from *Arthrobotrys* sp. CX1 capable of synergistically hydrolyzing cellulose with cellulases

**DOI:** 10.1186/s40643-021-00446-7

**Published:** 2021-09-24

**Authors:** Rong Li, Yunze Sun, Yihao Zhou, Jiawei Gai, Linlu You, Fan Yang, Wenzhu Tang, Xianzhen Li

**Affiliations:** School of Biological Engineering, Dalian Polytechnic University, Gangjingqu, Dalian, 116034 China

**Keywords:** Cellulose depolymerization, Crystalline region, Expansin-like protein

## Abstract

A novel expansin-like protein (CxEXL22) has been identified and characterized from newly isolated *Arthrobotrys* sp. CX1 that can cause cellulose decrystallization. Unlike previously reported expansin-like proteins from microbes, CxEXL22 has a parallel β-sheet domain at the N terminal, containing many hydrophobic residues to form the hydrophobic surface as part of the groove. The direct phylogenetic relationship implied the genetic transfers occurred from nematode to nematicidal fungal *Arthrobotrys sp*. CX1. CxEXL22 showed strong activity for the hydrolysis of hydrogen bonds between cellulose molecules, especially when highly crystalline cellulose was used as substrate. The hydrolysis efficiency of Avicel was increased 7.9-fold after pretreating with CxEXL22. The rupture characterization of crystalline region indicated that CxEXL22 strongly binds cellulose and breaks up hydrogen bonds in the crystalline regions of cellulose to split cellulose chains, causing significant depolymerization to expose much more microfibrils and enhances cellulose accessibility.

**Supplementary Information:**

The online version contains supplementary material available at 10.1186/s40643-021-00446-7.

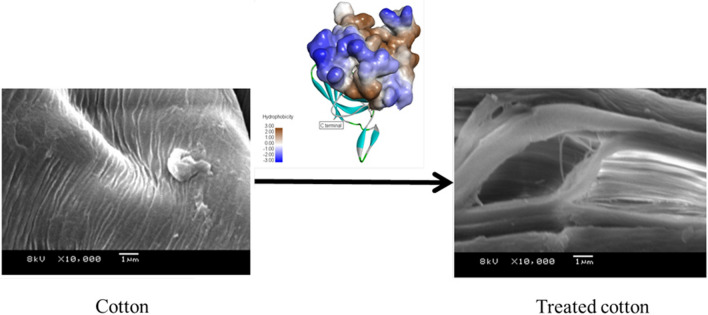

## Introduction

Cellulose is a polymer consisting of glucose line linked by β-1,4 bonds and contains crystalline region and amorphous region. Many studies in the past 50 years focused on the dissolution and degradation of cellulose as the renewable biomass and biomaterial (Guo et al. 2018; Ragauskas et al. 2006). However, the conversion of cellulose into fuel is still limited because of its physical properties, especially the degree of polymerization, crystallinity and accessibility (Arantes and Saddler 2010; Wilson 2009). The cellulose polymerization is formed by the agglomeration of microfibrils (dimeter around 10 nm, consisting of cellulose chains). The intermolecular and intramolecular hydrogen bonds tightly packed cellulose chains result in various ordered crystalline arrangements, forming cellulose crystalline region with high crystallinity. The cellulose accessibility is the most important factor for the rate and extent of cellulose hydrolysis, reflecting the available area for aqueous solution and cellulase to accessing and hydrolyzing cellulose chains, which is strongly influenced by polymerization and crystallinity (Arantes and Saddler 2010; Lindman et al. 2010). Thus, cellulose depolymerization and decrystallization must be improved to promote cellulose accessibility (Arantes and Saddler 2010; Lindman et al. 2010).

Ionic liquid and traditional organic solvents have been used in cellulose decrystallization and depolymerization. However, their application is limited by their environmental hazard, recovery problem and high cost (Rinaldi et al. 2008; Tadesse and Luque 2011; Chen et al. 2015). Enzymatic degradation is an environmental-friendly process of accessing and binding the cellulose chain to hydrolyze and diffuse cellulose (Sharma et al. 2019; Ravindran and Jaiswal 2015). Some accessory proteins, such as lytic polysaccharide monooxygenase (LPMO), expansin, expansin-like protein, swollenin and loosenin, can disrupt and lytic insoluble microfibril polymerization as cellulose pretreatment (Wilson 2012; Liu et al. 2015). These accessory proteins exhibit synergism with traditional cellulase to improve the cellulose degradation but cannot hydrolyze cellulose directly (Wilson 2012; Liu et al. 2015; Sabbadin et al. 2018). LPMOs produce several gaps on the surface of cellulose by oxidation, which promotes the accessibility of the cellulase to boost cellulose degradation (Villares et al. 2017).

Expansin is a wall-loosening protein obtained from plant. Various expansin-like genes with similar sequence homology and functions as expansin have been found in different microbes and other organisms that live in soil or produce cellulose (Liu et al. 2015; Cosgrove 2000; Georgelis et al. 2015). Recent reviews have detailed their discovery, classification, phylogenetic distribution and evolution. In addition, expansin-like from microbes show some similarity to expansin from plant: (i) these proteins loosen hydrogen bonds between polysaccharide chains without detectable hydrolytic activity; (ii) these proteins contain a double –Ψβ-barrel fold (DPBB) domain D1 at N-terminal and a β sandwich fold domain D2 at C-terminal; and (iii) these proteins have an acidic optimum to extension activity. These points are further supported by the fact that expansin-like proteins from microbes have evolved from a plant ancestor by horizontal gene transfer (HGT) (Nikolaidis et al. 2013). However, the expansin-like protein derived from nematodes is likely an example of independent evolution. Nematodes secrete a complex of hydrolytic enzymes to overcome the barrier presented by their host cell wall, which aid in root penetration and migration. The expansin-like protein from nematodes has a GH45 domain at its C-terminus, a structure indicative of separate evolutionary origin, which is inconsistent with canonical expansins (Liu et al. 2015; Sabbadin et al. 2018; Georgelis et al. 2015; Cosgrove 2015, 2017). Moreover the domain D1 of expansin and expansin-like protein resembles the catalytic domain of the GH45 without catalytic residues that is normally required for GH45 hydrolytic activity. The domain D2 forms a nearly flat surface populated with aromatic residues, resembling some carbohydrate-binding modules (CBM) to bind cellulose (Liu et al. 2015; Georgelis et al. 2015).

*Arthrobotrys sp*. CX1 is a nematicidal fungal strain that gelatinizes the filter paper cellulose. It was isolated and identified from soil sample with the rotted wood (Lan et al. 2016). It can gelatinize the filter paper cellulose causing decrystallization with high water imbibition, but cannot degrade cellulose. Thus, the cellulose decrystallization and depolymerization by *Arthrobotrys* sp. CX1 can facilitate water penetration in the cellulose but not cellulose degradation. In this work, we obtained a novel expansion-like protein (CxEXL22) from *Arthrobotrys* sp. CX1 that can cause cellulose decrystallization for the first time. Differing from proteins previously reported from microbes, CxEXL22 is composed of a parallel β-sheet domain at the N terminal and a DPBB expansin domain at the C terminal. The phylogenetic relationship showed the CxEXL22 was directly grouped with expansin-like proteins from nematode. It was indicated the expansin-like protein genetic transfers occurred from nematode to nematicidal fungal *Arthrobotrys sp*. CX1. The parallel β-sheet domain of the CxEXL22 forms the hydrophobic surface to bind the cellulose, although the domain is different from the β-sandwich fold domain D2 of the previous reports indicated expansin-like proteins. CxEXL22 was expressed and biochemically characterized. The rupture of the crystalline regions was examined with X-ray diffraction (XRD), scanning electron microscopy (SEM) and Fourier transform infrared spectrometer (FTIR) after the pretreatment with CxEXL22. Results suggested that CxEXL22 had a potential application in constructing unique nanocellulosic structures because of its capacity to induce cellulose depolymerization.

## Materials and methods

### General information

All chemical reagents in this study were obtained from commercial supplier Sinopharm Chemical Reagent Co. Ltd., Shanghai, China. Cellulosic substrates and commercial cellulase were purchased from Sigma-Aldrich, Mo, USA. Phosphoric acid-swelling cellulose (PASC)) was prepared from Avicel PH -101 (Kuo and Lee 2009). Fungal strain of *Arthrobotrys* sp. CX1 was isolated from the campus of Dalian Polytechnic University, China, that was cultured as previously described (Lan et al. 2016). *Pichia pastoris* X-33 and vector pPICαA were purchased from Invitrogen.

### Analysis and cloning of CxEXL22

The sequences of 15 full-length expansins and expansin-like proteins from different microbes, plants and nematode were aligned and a neighbor-joining phylogenetic tree was constructed using Molecular Evolutionary Genetics Analysis (MEGA) 6.0 software (Tamura et al. 2013). A conserved domain was identified using the Conserved domain Database. Total RNA from *Arthrobotrys* sp. CX1 was extracted using Trizol method that was grown in the presence of the filter paper (Lan et al. 2016). The CxEXL22 cDNA sequence was amplified from a sample of total RNA of *Arthrobotrys* sp. CX1 by reverse transcription coupled-PCR with the following primers: CxEXL22-T forward primer 5′-GACATGTTACAAAAGCGGG-3′, CxEXL22-T reverse primer 5′-TTAGACGTAATCCCAGGTG-3′. The 633-bp PCR fragment was purified and cloned into the pMD 18-T Vector (Takara) resulting in pMD 18-*CxEXL22*, and its sequence was confirmed. The pMD 18-*CxEXL22* as template was amplified by restriction-free cloning using the following primers: 5′-CTAAAGAAGAAGGGGTATCTCTCGAGAAAAGACATGTTACAAAAGCGGG-3′and5′-GAGTTTTTGTTCTAGAAAGCTGGCGGCCGCTTAGACGTAATCCCAGGTG-3′ and constructed with pPICZαA (Van Den Ent and Löwe 2006). Recombinant plasmid pPICZαA-*CxEXL22* was transformed into the host *Escherichia. coli* DH10B and its sequence was confirmed by restriction analysis and DNA sequencing. The nucleotide sequence of CxEXL22 has been deposited in the Genbank database under submission MN138044, which is without the signal peptide.

### Modeling the structure of CxEXL22

The CxEXL22 was used for homology modeling using the Phyre2 web server (Kelley et al. 2015). The structure of CxEXL22 is modeled based on EXLX1 [PDB ID: 3D30] and Clavibacter michiganensis expansin [PDB ID: 4JCW]. The sequence identity is 26% between CxEXL22 with EXLX1, and it is 36% between CxEXL22 with Clavibacter michiganensis expansin.

### Expression and purification of CxEXL22

*P. pastoris* X-33 was electrically transformed with linearized vector pPICZαA-*CxEXL22*, and transformants were selected by Zeocin (100 µg/mL). The *CxEXL22* gene in the transformants was confirmed by PCR using yeast genomic DNA as template to be transformed into *P. pastoris* X-33. The CxEXL22 protein was expressed in *P. pastoris* X-33 in accordance with the manufacturer’s protocols (Invitrogen).

The crude supernatant was collected by centrifugation at 8000 rpm for 5 min after cultured. The recombinant CxEXL22 was purified by concentrating the supernatant 15-fold by ultrafiltration at 4000 rpm for 40 min using centrifugal filter devices (exclusion size 10 kDa, Millipore), and then applying it to a HisTrap HP column (5 mL, GE Healthcare) pre-equilibrated with phosphate buffer (20 mM pH 6.0). The fractions were collected and checked by SDS-PAGE.

### CxEXL22 and commercial cellulase synergistically acted on substrates

CxEXL22 was used to measure activity with final concentrations of 100 mM buffer, 5 mg substrate, 5 μg CxEXL22 and 0.3 U commercial cellulase in a 5 mL reaction at pH 6 for 40 h at 30 °C with 160 rpm. According to the literature, the commercial cellulase was loaded into the reaction mixture (Lee et al. 2010). The concentration of the released reducing sugar was determined using the DNS method described previously (Zhang et al. 2009). All assays were performed in triplicate.

The reaction was treated at different temperatures (30, 40, 50, 60, or 70 °C) to determine the effect of temperature on CxEXL22. The reaction was terminated and reducing sugars were determined as explained above. For the whole range of buffer, the reducing sugar was determined in the reaction at the pH range of 3.0–7.0. The reaction was treated at different cellulase loading (0.05–0.45 U/ml) to determine cellulase loading to affect the synergetic action of CxEXL22.

### Analysis with XRD, IFTR and SEM

CxEXL22 was used to measure activity with final concentrations of 100 mM buffer, 5 mg substrate(cotton, filter paper and Avicel), 5 μg CxEXL22 in a 5-mL reaction at pH 6 for 48 h at 30 °C with 160 rpm. Control experiments without CxEXL22 or using H_2_O were also performed under the same conditions as mentioned above. For microscopic observations, the samples were washed three times by ultrasonic and then dried to completely remove all residual solvent. Then the substrates were visualized by SEM (JSM-7800F and JSM-6460LV, JEOL, Japan). The changes in chemical bonds and functional groups were detected by FTIR (Spectrum two, PerkinElmer, China) and changes in cellulose crystallinity and structure were determined by XRD (XRD-7000S, Shimadzu, Japan) as described previously (Lan et al. 2016).

## Results and discussion

### Sequence analysis of an expansin-like protein from *Arthrobotrys* sp. CX1

**Fig. 1:**
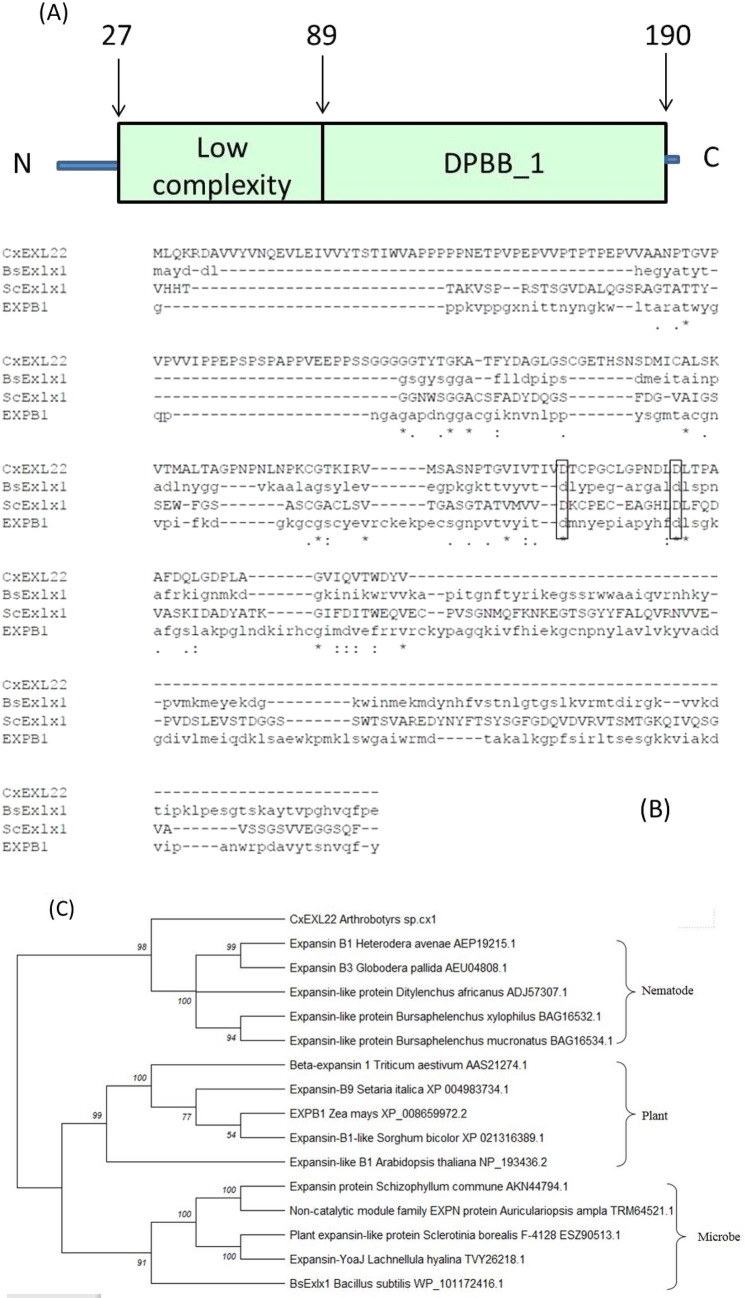
Sequence and phylogenetic analysis of CxEXL22. **A** The predicted domain organization of CxEXL22; **B** sequence alignment of CxEXL22, BsEXLX1 from *Bacillus subtilis* (GenBank: AHH91628.1), EXPB1 from *Zea mays* (NCBI: XP_008659972.2), and ScExlx1 from *Schizophyllum commune* (GenBank: AKN44794.1); C phylogenetic tree of CxEXL22

*Arthrobotrys* sp. CX1 was isolated from a soil sample with rotted wood collected from the campus of Dalian Polytechnic University, China, which can disrupt cellulose chains to make the filter paper translucent (Lan et al. 2016). Our interest is to reveal fungal-derived enzymes responsible for this novel cellulolytic and cellulose destruction activity. On the basis of sequences analysis from cDNA library of *Arthrobotrys* sp. CX1, one sequence was termed “*CxEXL22*”, which encoded a protein of 193 amino acid residues, containing the low complexity region (residues 27–89) and the DPBB region (residues 89–190), as predicted using the SMART server (Letunic et al. 2021) (Fig. 1A). The DPBB Domain of *CxEXL22* was found to have sequence similarity with expansin-like proteins from fungal species *Schizophyllum commune* H4-8 [NCBI:XP_003029770.1] 47.12%, *Heterobasidion irregulare* TC32-1 [NCBI:XP_009552773.1] 46.15% and *Gigaspora rosea* [GenBank: RIB28841.1], annotated as an expansin-like family protein. It has been identified that these crucial residues D71 and D82 in BsEXLX1 for the expansin activity (Kerff et al. 2008). These key residues are all completely conserved in CxEL22, suggesting the domain DPBB in CxEL22 has the function same as the domain DBPP of expansin-like proteins (Fig. 1B). CxEXL22 contains DPBB expansin domain at C terminal, which is different from proteins previously reported from microbes. Most of the expansin-like proteins from microbes reported the special DPBB domain at the N terminal. However, those from nematode contain the DPBB expansin domain at the C terminal (Georgelis et al. 2015; Nikolaidis et al. 2013; Cosgrove 2015).

The sequences of the expansin-related proteins were extracted and generated a phylogenetic tree, containing proteins from plants, microbe and nematode. An analysis of the phylogenetic relationship among these proteins showed that the sequences were separated into three clades (Fig. 1C). According to previous research of evolution of expansin-like proteins, these non-plant expansin-like proteins from bacteria and fungi originated in plant and transferred from plant to microbe by horizontal gene transfer, which revealed the evolution of plant and microbe species (Nikolaidis et al. 2013). Interestingly, the phylogenetic tree showed that the CxEL22 was directly grouped with expansin-like proteins from nematode. CxEL22 is identified from *Arthrobotrys* sp. CX1, which is a nematicidal fungal strain. The result strongly suggested that the expansin-like protein genetic transfers occurred from nematode to nematicidal fungal *Arthrobotrys* sp. CX1 (Additional file 1).

### Homology modeling of CxEXL22

**Fig. 2:**
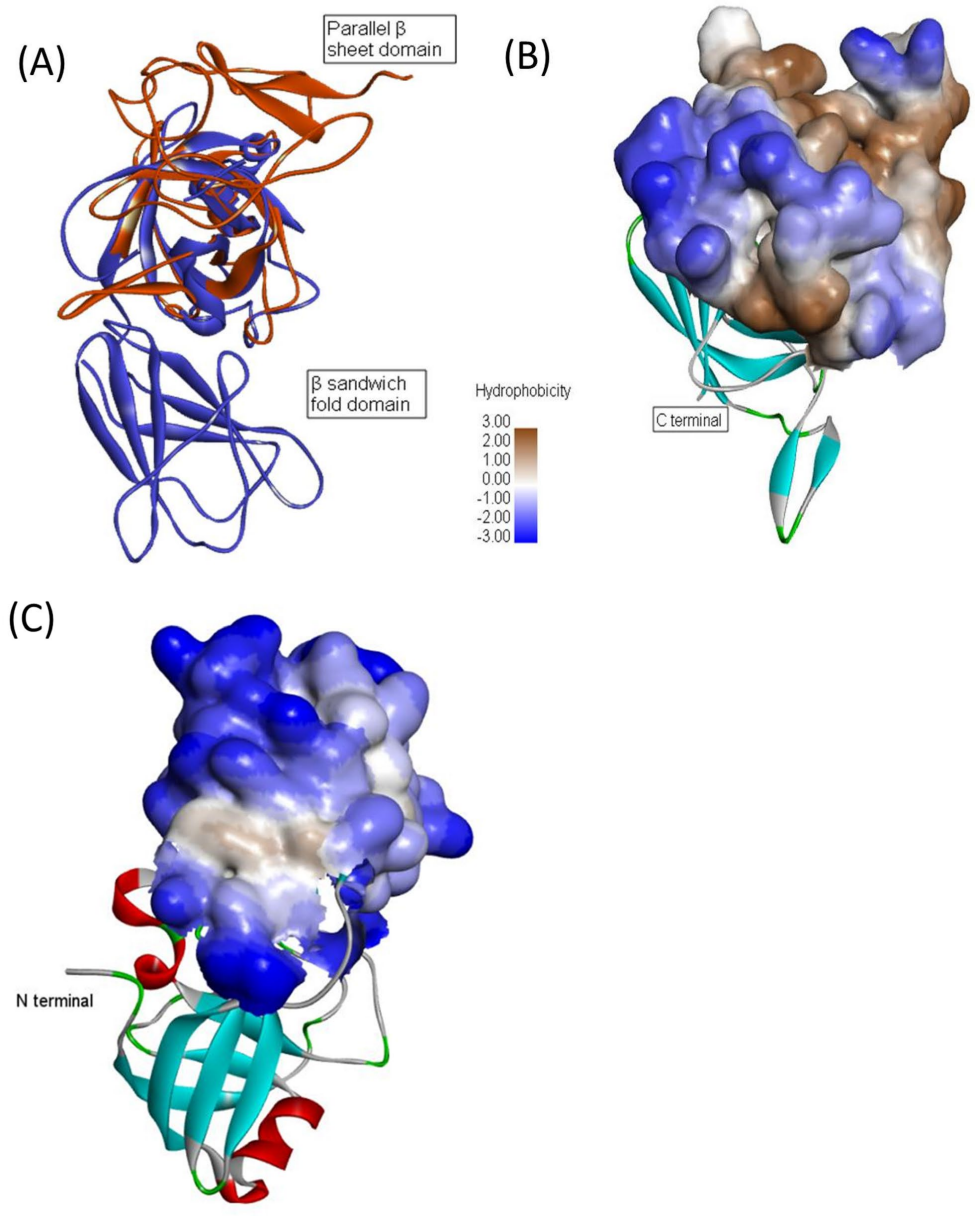
The predicted structure model of CxEXL22: **A** overall structures superimposition of the predicted CxEXL22 modeling (orange) and BsExl (PDB:3D30, blue); **B** the hydrophobic groove surface of parallel β sheet domain; C the hydrophobic surface of BsExl

Most of expansins and expansin-like proteins consist of two domains (D1 and D2). The domain D1 is named DPBB domain to catalysis and the domain D2 is a β-sandwich fold to bind, resembling CBM (Liu et al. 2015; Georgelis et al. 2015) The CxEXL22 was used for fold recognition using the Phyer web server. Unlike the previously reported structure of expansin-like protein, the CxEXL22 protein was shown to have the parallel β-sheet domain at the N terminal and a DPBB domain at the C terminal (Fig. 2A). Previous reports indicated the expansin-like proteins to bind the cellulose via the hydrophobic surface of the domain D2, consisting of hydrophobic interactions of three linearly arranged aromatic residues (Liu et al. 2015; Cosgrove 2000; Georgelis et al. 2015). The parallel β-sheet domain of the CxEXL22 protein is different from that of the domain D2 of expansin-like protein. By comparing the β-sandwich-fold domain and the parallel β-sheet domain, both contain hydrophobic residues to form the hydrophobic surface, the β-sandwich-fold domain is flat surface and less hydrophobic area, while the parallel β-folded domain appears as a groove with more hydrophobic surface (Fig. 2B, C). The model suggests that the CxEXL22 binds to the cellulose through the groove on the hydrophobic surface of the parallel β-sheet domain, which is more effective than the β-sandwich-fold domain to contact the hydrophobic surface of cellulose microfibrils.

### Heterologous expression of CxEXL22 in* P. pastoris*

**Fig. 3:**
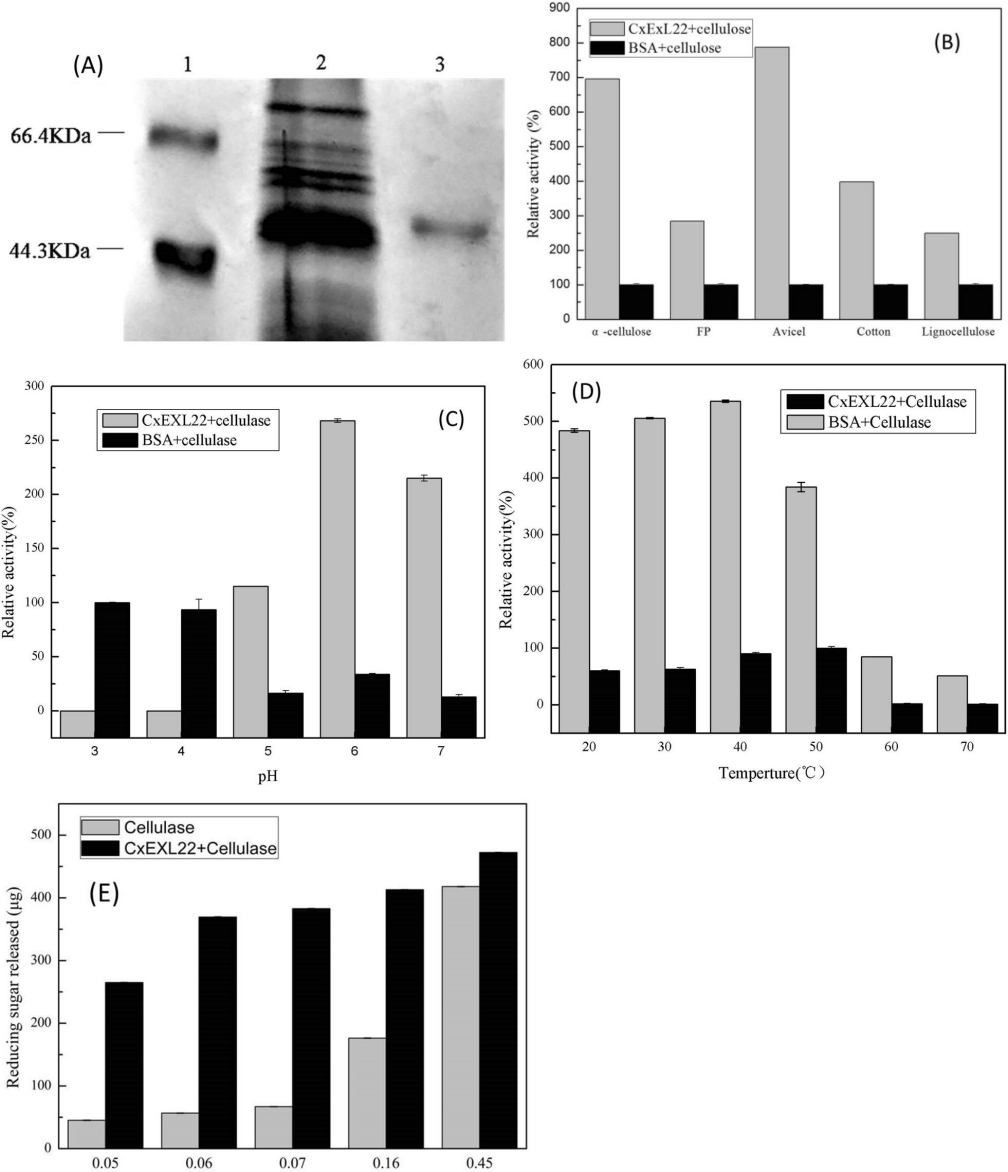
Characterization of CxEXL22. A SDS-PACE for the expression of CxEXL22; B synergistic effect of CxEXL22 and commercial cellulase on cellulose hydrolysis; C effect of pH; D effect of temperature; E effect of cellulase loading on synergism

The recombinant CxEXL22 was heterologously expressed in *Saccharomyces cerevisiae* and purified in one step with His-tag from culture supernatants to the more than 90% purity. Its molecular weight was calculated as approximately 50 kDa by SDS-PAGE (Fig. 3A). DNA sequence analysis of the open reading frame revealed that it encoded a protein of 193 amino acid residues and a calculated mass of 21.7 kDa. This suggested the occurrence of the post-translational modification, especially glycosylation. The CxEXL22 were predicted N-glycosylation of Asn on residue 55 and 147 by the NetNGlyc 1.0 Server. In previous reports heterologously expressed expansin-like proteins also exhibit glycosylation in fungal hosts (Wang et al. 2014; Liu et al. 2014).

### Synergistic effect of CxEXL22 in cellulose hydrolysis

Expansin-like proteins hydrolyze the hydrogen bond between poly chains to rupture the crystalline, but its hydrolytic activity cannot be detected alone (Ravindran and Jaiswal, 2015; Wilson 2012). The reducing sugar was not detected with the treated filter paper by CxEXL22 alone after 48 h. It was found that the released reducing sugar was increased by 2.4-fold when the filter paper was treated with a mixture of CxEXL22 and cellulase for 12 h than that with both bovine serum albumin (BSA) and cellulase. It suggested that the CxEXL22 could synergistically hydrolyze cellulose with cellulose. Such synergistic action with the commercial cellulase was investigated using different substrates including microcrystalline cellulose (Avicel), cotton, filter paper, lignocellulose and α-cellulose. As shown in Fig. 3B, CxEXL22 could significantly increase the efficiency of cellulose hydrolysis. The hydrolytic efficiency of Avicel was enhanced by 7.9 times when treated with CxEXL22, whereas the relative activity of the treated filter paper and lignocellulose was only increased by 2.4 and 2.2 times, respectively. Avicel is a microcrystalline cellulose with a higher crystallinity than that of filter paper composed of both amorphous regions and crystalline fibers. It was reported that the expansin-like proteins show strongly synergism activity to filter paper than Avicel (Wang et al. 2014; Georgelis et al. 2014). Thus the CxEXL22 showed high specificity on crystalline cellulose, affected crystallinity and accessibility of cellulose to produce a synergistic effect with cellulase for cellulose hydrolysis.

### Optimization of CxEXL22 hydrolyzing cellulose

The optimum hydrolysis conditions of CxEXL22 for disrupting the crystalline region were determined. As shown in Fig. 3C, cellulase gave a high activity at pH 3.0 and 4.0 in the control with cellulase and bovine serum albumin, which is consistent with the optimum pH for the cellulase from *Aspergillus niger* (Hurst et al. 1977). However, the highest activity was obtained at pH 6.0 when CxEXL22 synergistically cooperated with cellulase, which moved away from an original pH. The pH dependence of CxEXL22 differed from those of previously reported and was inconsistent with the acid growth theory (Cosgrove 2000). In various plants, the plant cell wall grows faster when the wall pH is lowered below 5.5 and this phenomenon is known as acid growth (Dünser and Kleine-Vehn 2015). Expansins were isolated and identified to act as a catalyst to acid growth (Cosgrove 2000; Nakatani et al. 2013). This finding indicated that expansins had an acidic optimum to promote wall extension, which is similar to expansin-like proteins from microbes (Kim et al. 2009; Georgelis et al. 2014). It was of interest that no cellulase activity was detected to the CxEXL22-treated substrate at pH 3.0–4.0. Binding capability to the substrate depends on the pH of the incubation buffer (Liu et al. 2015). Expansins and cellulose compete for the limited binding sites on cellulose substrates (Kim et al. 2009; Liu et al. 2015). The result indicated the CxEXL22 may strongly bind to the polysaccharide substrate at acidic pH to seize the limited binding sites on cellulosic substrates, even completely inhibit the binding of cellulase. However, the CxEXL22 cannot depolymerize cellulose to expose much more microfibrils and enhances cellulose accessibility, resulting in cellulase cannot bind and hydrolase cellulose.

The hydrolysis activity of cellulase was set at 50 °C as the standard that is the reported optimal temperature (Hurst et al. 1977). However, the maximum synergistic activity of CxEXL22 with cellulase was achieved at 40 °C, and it was eightfold greater than that of control under the same reaction temperature (Fig. 3D). When the temperature was raised to 70 °C, it can still synergistically increase by twofold for cellulase activity although CxEXL22 activity was markedly lowered. This result indicated that CxEXL22 could be applied at a wide range of temperatures.

The cellulase loading is important for the synergism of expansin and cellulase to hydrolysis cellulose. As shown in Fig. 3E, the amount of reducing sugar released by cellulose hydrolysis increased significantly with the increase of the cellulase concentration from 0.05 to 0.45U/ml. But with the increase of cellulase loading, the synergistic effect became insignificant, which is consistent with the synergism of cellulase and BsEXLX1 (Kim et al. 2009). The synergistic effect is most significant at the low cellulase loading. Expansins and cellulase compete for the limited binding sites on substrates, resulting in a highly synergistic effect that occurs only at low concentrations of cellulase. Such low sugar yields in response to low cellulase loading are common when characterizing synergism between proteins and cellulases with CBM (Kim et al. 2009, 2014).

### Cellulose decrystallization by CxEXL22

**Fig. 4:**
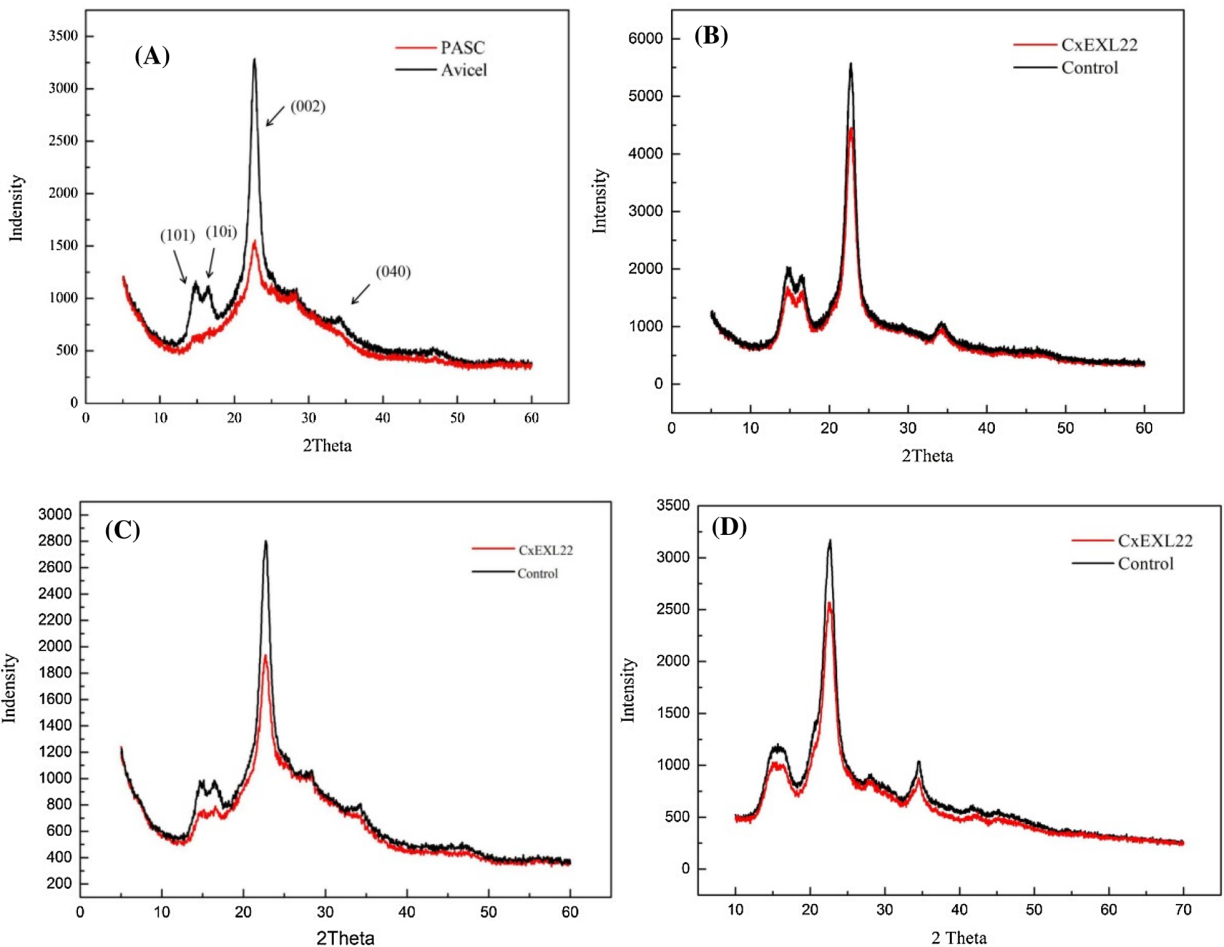
X-ray diffraction spectrum of different cellulose substrates. **A** Phosphoric acid-swelling Avicel, **B** cotton, **C** filter paper and **D** Avicel

Crystallinity index was examined by XRD to evaluate the destruction of CxEXL22 on cellulose crystalline region. The crystallinity index was calculated with the height of four crystalline peaks based on the amorphous standard from XRD data (Park et al. 2010). Acid hydrolysis of cellulose can cause a conversion of the crystalline region to the amorphous region, thus the XRD spectra of Avicel and Avicel swelled by phosphoric acid indicated the change in the crystalline region (Fig. 4A). XRD spectrum showed changes in four peaks named as 101, 10i, 002 and 040, respectively, which were similar to those have been assumed in other reports (Ciolacu et al. 2011; Kim et al. 2013). As shown in Fig. 4B–D, the reduction in the height of these four crystallization peaks was also observed in different cellulose substrates treated with CxEXL22. Therefore, the rupture of the ordered crystal lattice assignments of cellulose was transformed into amorphous region after CxEXL22 treatment. Cellulose crystallinity index was calculated based on comparing the background intensity at 2θ = 18° with the peak height of 002 at around 22.8°. The crystallinity index of Avicel was decreased by 21% after treated with acid and 3.5% after treated with CxEXL22, which indicated that the ordered structure of the crystalline region was disrupted (Table 1). Moreover, a strong decrease (about 8.5%) in the crystallinity degree was observed in the treated filter paper, presumably based on the accessibility of the filter paper, which contains much more terminal gaps between crystalline region and amorphous region for accessing by CxEXL22. In conclusion, CxEXL22 could significantly reduce cellulose crystallinity that will cause subsequent depolymerization of the crystalline region.

**Table 1:** Crystallinity index of CxEXL22-treated cellulose substrates, as determined by X-ray diffraction

Cellulose substrates	Crystallinity index (%)	
	Untreated	Treated
Phosphoric acid-swelling Avicel	75.35	54.32
Avicel	74.79	71.30
Cotton	82.04	78.12
Filter paper	70.64	62.12

**Fig. 5:**
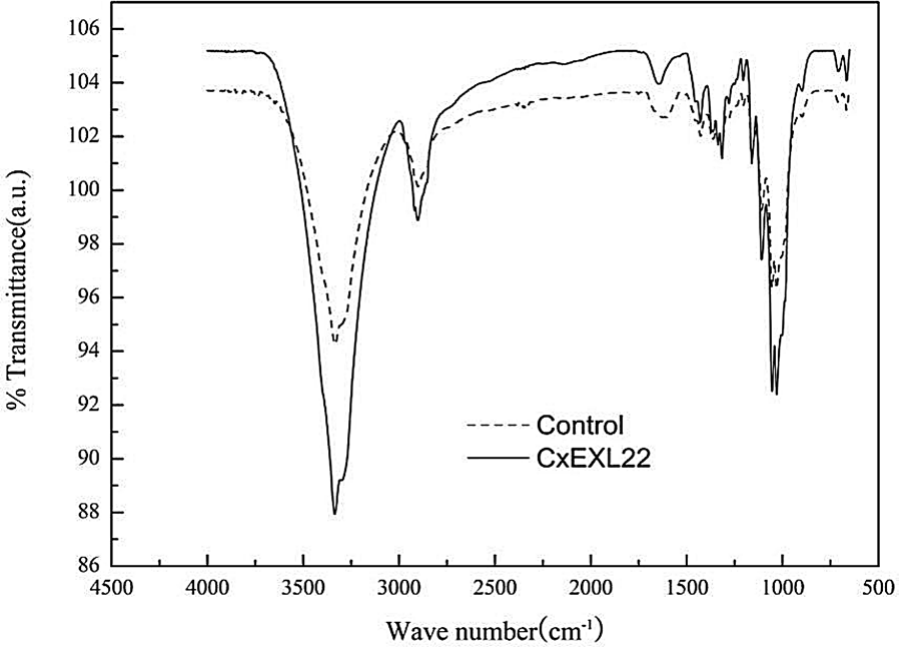
Comparison of Fourier-transform infrared spectra of Avicel treated with CxEXL22 with that of untreated Avicel

Structure of Avicel treated with CxEXL22 was analyzed by FTIR. The band near 3300 cm^−1^ can be attributed to O–H stretching vibrations (Ciolacu et al. 2011), which showed higher intensity than that of control (Fig. 5). It suggested the rupture of intra- and inter-molecular hydrogen bonds in the treated Avicel that resulted in the exposure of much more –OH groups (Ciolacu et al. 2011; Kim et al. 2013). The skeletal stretching of intramolecular C–OH is near 1030 cm^−1^. As shown in Fig. 5, the absorption of O–H stretching of the treated Avicel was intensified, which reflected the entrance of water molecules causing the exposure of more hydrogen bonds of networks in the crystalline region compared with the untreated Avicel. This result was well supported by the XRD observation above, which confirmed that the hydrogen bond between cellulose chains was hydrolyzed by CxEXL22 to expose more O–H groups between the chains and the O–H of the entered water molecules in polymers. This caused the decrystallization of the crystalline region leading to the depolymerization of cellulose.

### Cellulose depolymerization by CxEXL22

**Fig. 6:**
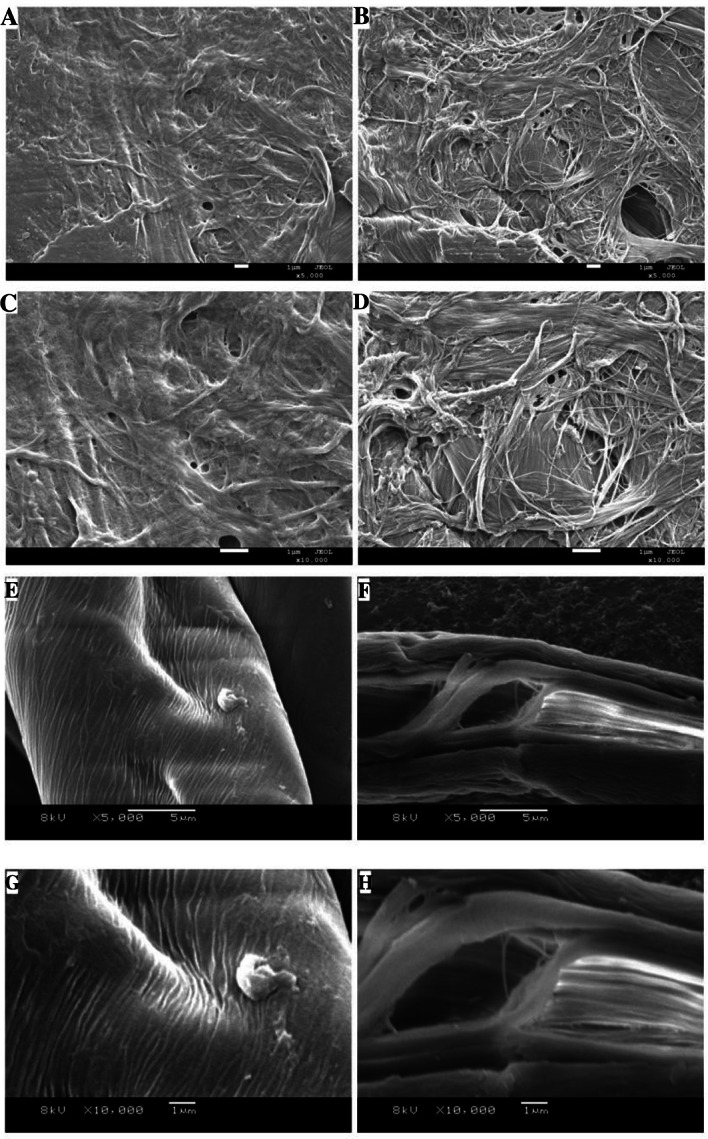
Scanning electron microscopy observation of different cellulose substrates: **A**, **C** ddH_2_O-treated filter paper at **A** 5000 × and **C** 10,000 × ; **B**, **D** CxEXL22-treated filter paper at **B** 5000 × and **D** 10,000 × ; **E**, **G** ddH_2_O-treated cotton at **E** 5000 × and **G** 10,000 × ; and **F**, **H** CxEXL22-treated cotton at **F** 5000 × and **H** 10,000 ×

To visualize the effect of CxEXL22 on cellulose polymer, microphotography of different polysaccharide substrates was performed using SEM. As suggested that macrofibril was the agglomeration of microfibrils (Chinga-Carrasco 2011), the surface of untreated filter paper showed macrofibrils with high density and overlapping, whereas the CxEXL22-treated filter paper showed dispersed microfibers (Fig. 6). Cotton fiber in the untreated control was smooth and flat, but the CxEXL22-treated cotton fiber showed microfibril bundles and loosened intermolecular surface between macrofibrils, which caused cellulose decrystallization and depolymerization. Such results suggested that the dense structure of crystalline region was broken and the looseness of cellulose was significantly increased after treated with CxEXL22. The binding of cellulose and expansin-like protein is entropy-driven triggered by the hydrophobic interactions between the cellulose and the hydrophobic flat in D2 (Georgelis et al. 2012). We deduced that the CxEXL22 binds to the macrofibrils by the hydrophobic surface of the parallel β-sheet domain, and slides on the cellulose chains to hydrolyze intermolecular and intramolecular hydrogen bonds tightly packed cellulose chains. Therefore, the agglomeration of microfibrils was distinctly decreased by CxEXL22 and the macrofibril was depolymerized to expose much more microfibrils and increase cellulose accessibility.

## Conclusion

The crystallinity and polymerization of cellulose is the key bottleneck to the efficiency of cellulose degradation. In this context, a novel expansin-like protein CxEXL22 caused cellulose decrystallization and exhibited effective synergistical catalytic activity against cellulose with cellulase. The direct phylogenetic relationship of CxEXL22 implied that genetic transfers occurred between nematode and nematicidal fungi. The result implied that CxEXL22 strongly binds to cellulose chain via the hydrophobic groove surface of parallel β-sheet domain and breaks up hydrogen bonds between cellulose chains. Consequently, CxEXL22 splits cellulose chains depolymerizing cellulose to expose much more microfibrils, thus enhancing cellulose accessibility.

### Supplementary Information

**Additional file 1**: **Table S1**. Expansin-like proteins from plants, nematodes and microbes used for the CxEXL22 phylogenetic analysis. **Fig. S1**. Conserved domains of CxEXL2. **Fig. S2**. The sequence alignment of CxEXL22 with modeling temple. **Fig. S3**. SEM images of (A) the original filter paper and (B) the phosphoric acid treated filter paper. **Fig. S4**. Structure alignment RMSD values of (1) the predicted structure of CxEXL22 protein with the Phyre2 web server (2) the predicted structure of CxEXL22 protein with the I-TASSER server and (3) the predicted structure of CxEXL22 protein with the Swiss-Model server

## Data Availability

The dataset (graphs and tables) supporting the conclusions of this article are available.
