# Supplementary material for: A novel decrystallizing protein CxEXL22 from Arthrobotrys sp. CX1 capable of synergistically hydrolyzing cellulose with cellulases
Source: Bioresour Bioprocess. 2021 Sep 24;8(1):90. doi: 10.1186/s40643-021-00446-7 (PMC10992334; doi:10.1186/s40643-021-00446-7)
Supplement: Supplementary file 1 — Additional file 1: Table S1. Expansin-like proteins from plants, nematodes and microbes used for the CxEXL22 phylogenetic analysis. Fig. S1. Conserved domains of CxEXL2. Fig. S2. The sequence alignment of CxEXL22 with modeling temple. Fig. S3. SEM images of (A) the original filter paper and (B) the phosphoric acid treated filter paper. Fig. S4. Structure alignment RMSD values of (1) the predicted structure of CxEXL22 protein with the Phyre2 web server (2) the predicted structure of CxEXL22 protein with the I-TASSER server and (3) the predicted structure of CxEXL22 protein with the Swiss-Model server [file 40643_2021_446_MOESM1_ESM.docx]

**Supplementary Information**

Contents: supplementary Table 1, supplementary figures 1 through 4

**A novel decrystallizing protein CxEXL22 from Arthrobotyrs sp. CX1 capable of synergistically hydrolyzing cellulose with cellulases**

Rong Li, Yunze Sun, Yihao Zhou, Jiawei Gai, Linlu You, Fan Yang, Wenzhu Tang, Xianzhen Li *

**Supplementary Table 1.** Expansin-like proteins from plants, nematodes and microbes used for the CxEXL22 phylogenetic analysis.

| Accession Number | Annotated activity [Organism] |
| --- | --- |
| [GenBank: MN138044] | CxEXL22 [*Arthrobotyrs sp.* CX1] |
| [GenBank: BAG16532.1] | expansin-like protein [Bursaphelenchus xylophilus] |
| [GenBank: BAG16534.1] | expansin-like protein [Bursaphelenchus mucronatus] |
| [GenBank: ADJ57307.1] | expansin-like protein [Ditylenchus africanus] |
| [GenBank: AEP19215.1] | expansin B1 [Heterodera avenae] |
| [GenBank: AEU04808.1] | expansin B3 [Globodera pallida] |
| [GenBank: AKN44794.1] | expansin protein [Schizophyllum commune] |
| [GenBank: AHH91628.1] | BsExlx1 [Bacillus subtilis] |
| [GenBank: TRM64521.1] | Non-catalytic module family EXPN protein [Auriculariopsis ampla] |
| [GenBank: ESZ90513.1] | plant expansin-like protein [Sclerotinia borealis F-4128] |
| [GenBank: TVY26218.1] | Expansin-YoaJ [Lachnellula hyalina] |
| [NCBI: XP_008659972.2] | EXPB1 [Zea mays] |
| [NCBI: XP_021316389.1] | expansin-B1-like [Sorghum bicolor] |
| [NCBI: XP_004983734.1] | expansin-B9 [Setaria italica] |
| [NCBI: NP_193436.2] | expansin-like B1 [Arabidopsis thaliana] |
| [GenBank: AAS21274.1] | beta-expansin 1 [Triticum aestivum] |


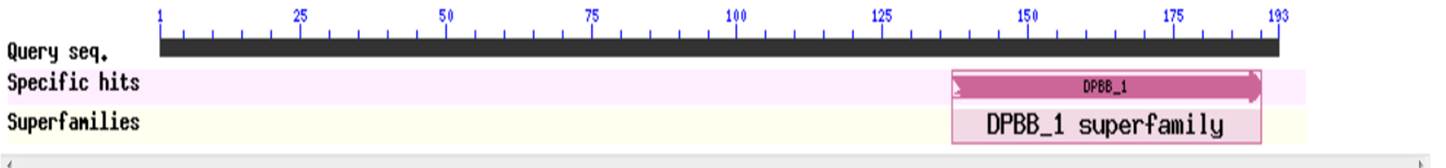


**Fig.S1.** Conserved domains of CxEXL2


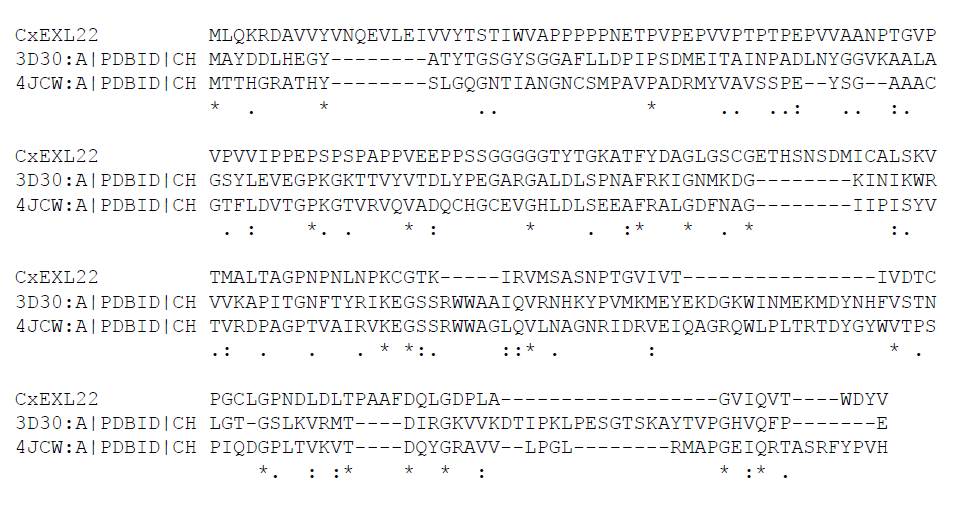


**Fig.S2.** The sequence alignment of CxEXL22 with modeling temple


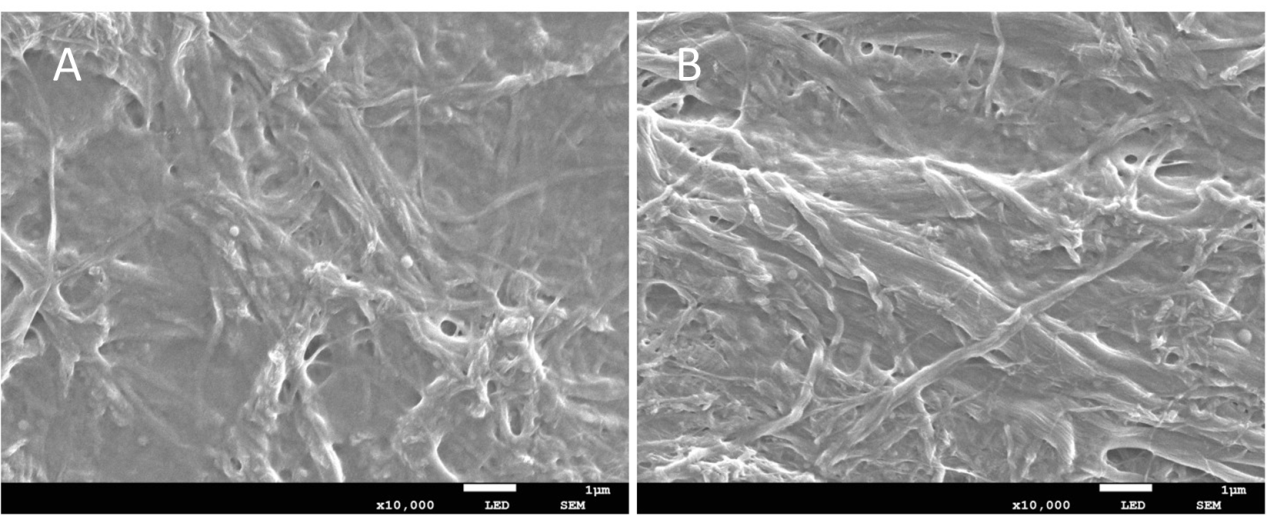


**Fig.S3.** SEM images of (A) the original filter paper and (B) the phosphoric acid treated filter paper


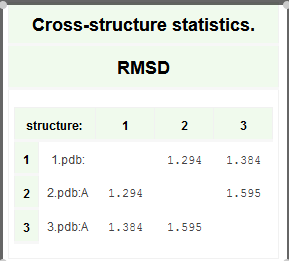


**Fig.S4.** Structure alignment RMSD values of (1) the predicted structure of CxEXL22 protein with the Phyre2 web server (2) the predicted structure of CxEXL22 protein with the I-TASSER server and(3) the predicted structure of CxEXL22 protein with the Swiss-Model server.
